# ER stress induces NLRP3 inflammasome activation and hepatocyte death

**DOI:** 10.1038/cddis.2015.248

**Published:** 2015-09-10

**Authors:** C Lebeaupin, E Proics, C H D de Bieville, D Rousseau, S Bonnafous, S Patouraux, G Adam, V J Lavallard, C Rovere, O Le Thuc, M C Saint-Paul, R Anty, A S Schneck, A Iannelli, J Gugenheim, A Tran, P Gual, B Bailly-Maitre

**Affiliations:** 1 INSERM, U1065, Equipe 8 « Complications hépatiques de l'obésité », Bâtiment Universitaire ARCHIMED, Nice, France; 2 Université de Nice Sophia Antipolis, Faculté de Médecine, Nice, France; 3 Centre Hospitalier Universitaire Nice, Hôpital l'Archet, Département Digestif, Nice, France; 4 Centre Hospitalier Universitaire Nice, Hôpital l'Archet, Département Biologie, Nice, France; 5 Institut de Pharmacologie Moléculaire et Cellulaire, CNRS, UMR7275, Valbonne, France

## Abstract

The incidence of chronic liver disease is constantly increasing, owing to the obesity epidemic. However, the causes and mechanisms of inflammation-mediated liver damage remain poorly understood. Endoplasmic reticulum (ER) stress is an initiator of cell death and inflammatory mechanisms. Although obesity induces ER stress, the interplay between hepatic ER stress, NLRP3 inflammasome activation and hepatocyte death signaling has not yet been explored during the etiology of chronic liver diseases. Steatosis is a common disorder affecting obese patients; moreover, 25% of these patients develop steatohepatitis with an inherent risk for progression to hepatocarcinoma. Increased plasma LPS levels have been detected in the serum of patients with steatohepatitis. We hypothesized that, as a consequence of increased plasma LPS, ER stress could be induced and lead to NLRP3 inflammasome activation and hepatocyte death associated with steatohepatitis progression. In livers from obese mice, administration of LPS or tunicamycin results in IRE1*α* and PERK activation, leading to the overexpression of CHOP. This, in turn, activates the NLRP3 inflammasome, subsequently initiating hepatocyte pyroptosis (caspase-1, -11, interleukin-1*β* secretion) and apoptosis (caspase-3, BH3-only proteins). In contrast, the LPS challenge is blocked by the ER stress inhibitor TUDCA, resulting in: CHOP downregulation, reduced caspase-1, caspase-11, caspase-3 activities, lowered interleukin-1*β* secretion and rescue from cell death. The central role of CHOP in mediating the activation of proinflammatory caspases and cell death was characterized by performing knockdown experiments in primary mouse hepatocytes. Finally, the analysis of human steatohepatitis liver biopsies showed a correlation between the upregulation of inflammasome and ER stress markers, as well as liver injury. We demonstrate here that ER stress leads to hepatic NLRP3 inflammasome pyroptotic death, thus contributing as a novel mechanism of inflammation-mediated liver injury in chronic liver diseases. Inhibition of ER-dependent inflammasome activation and cell death pathways may represent a potential therapeutic approach in chronic liver diseases.

Nonalcoholic fatty liver disease (NAFLD) has become the most common form of chronic liver disease, currently affecting 20–30% of the general population and 75–100% of obese individuals.^1^ The spectrum of NAFLD is wide ranging: from hepatic steatosis to nonalcoholic steatohepatitis (NASH) and hepatocellular carcinoma. Hepatic steatosis is characterized by triglyceride accumulation in hepatocytes and follows a benign non-progressive clinical course. Nonalcoholic steatohepatitis (NASH), a progressive form, is defined as a combination of lipid accumulation, hepatocyte death, inflammation and fibrosis. As the probability of developing advanced fibrosis and hepatocellular carcinoma^2^ is significantly greater in patients with steatohepatitis than in those with simple steatosis, it is important to elucidate the mechanism underlying the progression from steatosis to steatohepatitis.

The endoplasmic reticulum (ER) stress response has been linked to obesity, type 2 diabetes and liver cancer.^3, 4^ Under stress conditions, the ER initiates the unfolded protein response (UPR) to restore homeostasis. The UPR involves three transmembrane sensors: inositol-requiring enzyme 1 (IRE1*α*), PKR-like ER kinase (PERK) and activating transcription factor (ATF6).^5^ Each pathway culminates in the transcriptional regulation of gene expression, which first seeks to reestablish ER homeostasis. Failure of the UPR to decrease ER stress leads to apoptosis, notably via CHOP, a pro-apoptotic transcription factor whose expression is highly induced by ER stress.^6^

Increased activation of the ER stress response has been reported in obese mice and humans.^3, 4, 7, 8^ Obesity results in liver ER stress, which promotes insulin resistance and hepatosteatosis through the IRE1*α* branch.^3^ Moreover, PERK and IRE1*α* can regulate lipid stores in the liver, enforcing the hepatic metabolic disorders associated with obesity.^9, 10^ It is well established that apoptosis and inflammation are increased in patients with NASH, correlating with histological severity. Because the ER stress response is a critical mediator of inflammation, apoptosis and insulin resistance, it could have a central role in the progression from steatosis to NASH. However, the evidence for activation of hepatic ER stress in patients with NASH needs to be clarified. Gonzalez-Rodriguez *et al.*^11^ observed that NASH patients displayed more elevated ER stress markers, namely CHOP and GRP78, reinforcing the notion that enhanced ER stress within liver cells may be relevant in the progression from steatosis to NASH.^12, 13, 14^

In addition, while studies indicated a contribution of NF-*κ*B in the inflammatory responses triggered as a consequence of hepatic ER stress associated with NASH,^15^ the potential interplay between ER stress and inflammasome engagement has yet to be explored in NASH progression. The NLRP3 inflammasome is a multi-protein complex which instigates the inflammatory response and contributes to insulin resistance. The NLRP3 inflammasome senses obesity-associated danger signals, namely endotoxin (LPS),^16^ hyperglycemia and free fatty acids (FFAs), and mediates caspase-1-dependent maturation of the proinflammatory cytokines interleukin-1*β* (IL-1*β*) and IL-18.^17^ Importantly, increased plasma LPS levels have been detected in mice models of NAFLD^18^ and in humans with NASH.^19, 20, 21^ Studies have suggested that the NLRP3 inflammasome may have a deleterious role in steatosis and NASH pathogenesis. Indeed, a deficiency in *caspase-1*, *Nlrp3* or *ASC* in mice results in protection from high-fat diet (HFD)-induced steatosis and insulin resistance.^16, 22, 23^ Similarly, a deficiency in *IL-1β*, *IL-1 Receptor* or *TLR4*^24^ protects mice from methionine- and choline-deficient (MCD) diet-induced steatohepatitis. Moreover, the NLRP3 inflammasome also triggers pyroptosis, a form of programmed cell death. Pyroptosis is defined as a caspase-1 or caspase-11-dependent cell death subroutine that is associated with the generation of pyrogenic mediators such as IL-1*β* and IL-18.^25, 26^ Therefore, the NLRP3 inflammasome could be a major cause of cell death and inflammation in NASH progression.

A ‘two hit' mechanism has been proposed to drive NASH pathogenesis.^27^ The first hit is associated with steatosis and sensitizes the liver to additional proinflammatory insults (second hit), such as LPS, which aggravate liver injury and contribute to the development of NASH.^28, 29^ We hypothesized that, as a consequence of increased plasma LPS, ER stress could be induced and lead to NLRP3 inflammasome activation and hepatocyte death associated with NASH. To address this issue, we explored *in vivo* whether the administration of LPS could trigger exaggerated hepatic ER stress signaling, and compared the response with that of tunicamycin, a chemical ER stress inducer, in steatotic livers from genetically obese (*ob/ob*) mice. We analyzed the potential benefit of TUDCA, an ER stress inhibitor, in the prevention and treatment of hepatic inflammation and death caused by an LPS challenge. We found that PERK and IRE1*α* pathways cooperate to activate CHOP, and that this appears to be a critical link between inflammasome activation and hepatocyte death in NASH. Importantly, the upregulation of transcripts of ER stress correlated with inflammasome priming and liver injury in NASH patients, which highlights their relevance in disease progression.

## Results

### TUDCA protects the liver from LPS-induced injury, apoptosis and inflammasome priming

**Figure 1:**
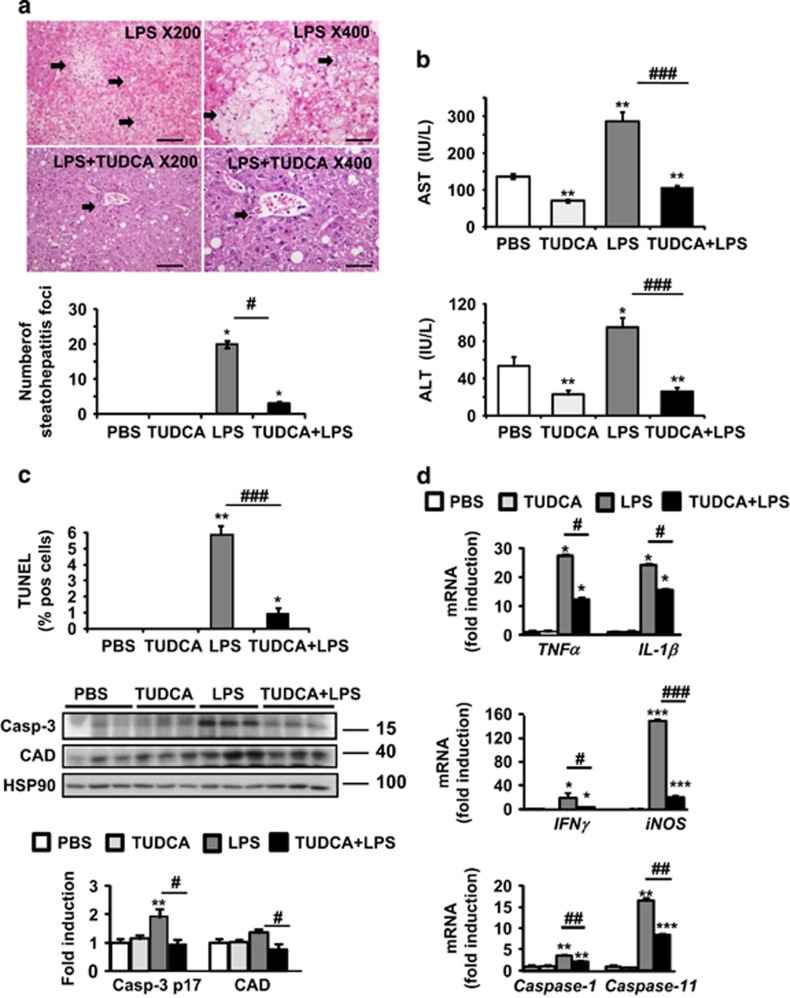
TUDCA protected against LPS-induced liver injury, apoptosis and inflammasome priming in *ob/ob* mice. TUDCA was injected intraperitoneally (500 *μ*g/g) for 5 days. An LPS challenge (2 *μ*g/g) was performed 6-h before killing. (**a**) Shown are photomicrographs of sections of murine liver stained with H&E (scale bar=50 *μ*m at × 200 or 25 *μ*m at × 400 magnification). The number of steatohepatitis foci (number of inflammatory foci in contact with ballooned hepatocytes, identified by arrows) was evaluated. (**b**) Serum AST and ALT transaminase levels were measured (*n*=9–12). (**c**) Apoptotic hepatocytes were visualized with TUNEL assay. The expression of active caspase-3 and CAD was evaluated in total liver lysates. Quantification was performed from the immunoblot analysis and expressed as fold induction (*n*=6). (**d**) Relative expression of hepatic *TNFα, IL-1β, caspase-1* and *caspase-11* mRNA (normalized to *36B4* mRNA). Data were expressed as fold induction (*n*=7). Data are expressed as mean±standard error of the mean. Statistical significance from controls is denoted by *^,#^*P*≤0.05, **^,##^*P*≤0.01, ***^,###^*P*≤0.001

In genetically obese mice (*ob/ob*) with severe steatosis and challenged with LPS, we investigated the *in vivo* effects of TUDCA treatment. Liver histological analysis revealed severe inflammation with many inflammatory foci and areas of cell death in LPS-injected mice compared with PBS-injected mice (Figure 1a). Five days of TUDCA treatment dramatically reduced the number of steatohepatitis foci (presence of inflammatory foci and ballooned hepatocytes) induced by LPS (Figure 1a). TUDCA treatment also resulted in partial resolution of hepatic steatosis (Figure 1a and Supplementary Figure S1A). As expected, inflammatory foci were absent in PBS- and TUDCA-injected mice (Supplementary Figure S1A). Consistent with these observations, serum levels of aspartate (AST) and alanine (ALT) aminotransferases were significantly lower in [TUDCA+LPS]-treated *ob/ob* mice compared with LPS-treated mice (Figure 1b). TUDCA-treated mice also displayed a reduction in AST and ALT levels compared with those receiving PBS. Thus, in *ob/ob* mice, LPS challenge induced NASH-like pathological features: ballooned hepatocytes, liver damage and inflammation. TUDCA treatment prevented these effects. After LPS injection, liver sections of TUDCA*-*treated mice also contained less TUNEL-positive hepatocytes compared with untreated animals. TUNEL staining showed that both apoptosis (nuclear fragmentation, Figure 1c) and necrosis (diffuse cytoplasmic staining, Supplementary Figure S1A) were significantly reduced after TUDCA treatment. Furthermore, the levels of active caspase-3 and substrate CAD were markedly reduced in [TUDCA+LPS]-treated mice in comparison with LPS-treated ones (Figure 1c). In accordance with inflammatory cell infiltration, the hepatic levels of *TNFα, IL-1β*, *IFNγ* and *iNOS* messenger mRNA were significantly increased in LPS-injected mice (Figure 1d). TUDCA treatment decreased the hepatic levels of these markers upon LPS challenge. Notably, IL-1*β* is matured by proinflammatory caspases (caspase-1 and caspase-11).^17^ Interestingly, the analysis of mRNA levels showed that *caspase-1* and *caspase-11* were significantly decreased in the livers of [TUDCA+LPS]-treated mice in comparison with LPS-treated ones (Figure 1d). We concluded that TUDCA treatment suppresses hepatocyte ballooning, apoptosis and inflammasome priming upon LPS challenge in *ob/ob* mice.

### TUDCA provides protection against LPS-induced ER stress and inflammasome pyroptotic death

**Figure 2:**
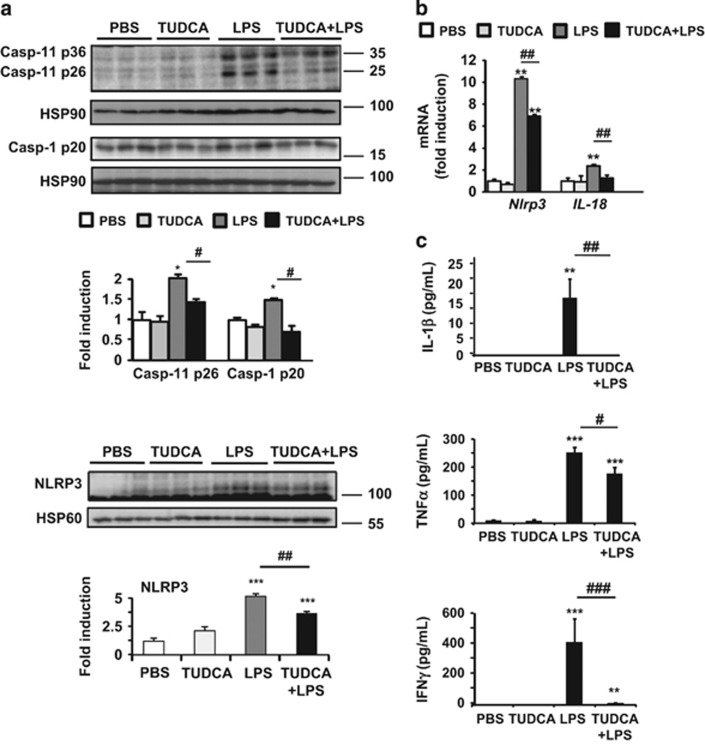
TUDCA treatment prevented hepatic activation of the NLRP3 inflammasome in *ob/ob* mice challenged with LPS. (**a**) Analysis of whole-liver samples from PBS-, TUDCA-, LPS- and [TUDCA+LPS]-treated mice. Immunoblot analysis of active caspase-11, active caspase-1 and NLRP3 protein levels are shown (*n*=4–6). (**b**) Real-time quantitative PCR analysis was performed to compare relative hepatic levels of *Nlrp3* and *IL-18* mRNAs. (**c**) Plasma cytokine levels were quantified for IL-1*β*, TNF*α* and IFN*γ* (*n*=7–9). Statistical significance from controls is denoted by *^,#^*P*≤0.05, **^,##^*P*≤0.01, ***^,###^*P*≤0.001

As TUDCA treatment prevented LPS-induced upregulation of *IL-1β, caspase-1* and *caspase-11* gene expression (Figure 1d) and TUNEL positivity (Figure 1c), we hypothesized that LPS could induce hepatic ER stress pyroptosis. As in apoptotic cell death, cells undergoing pyroptosis incur DNA damage and become positive in the terminal deoxynucleotidyl transferase dUTP nick-end labeling (TUNEL) assay. Also, Figure 2a shows that LPS-treated *ob/ob* mice exhibited marked increases in both active caspase-11 and -1, whereas TUDCA strongly prevented activation. As reported,^16^ hepatic steatosis was already associated with increased levels of both active inflammatory caspases in the liver. We observed that NLRP3 expression was induced upon LPS stimulation at the protein and mRNA levels, whereas it was decreased with TUDCA, as shown in Figures 2a and b, respectively. Accordingly, hepatic activation of another specific inflammasome substrate, *IL-18*, was reduced at the mRNA level (Figure 2b). Importantly, in agreement with increased caspase-11 and -1 activation, the serum levels of systemic mature IL-1*β* rose after LPS injury, whereas TUDCA treatment completely abolished IL-1*β* secretion in these mice (Figure 2c). TUDCA treatment also decreased the circulating levels of global inflammatory markers such as TNF*α*, IFNγ (Figure 2c), MCP-1 and IL-6 (Supplementary Figure S1B) in response to LPS. It should be acknowledged that the circulating inflammatory markers could be derived from adipose tissue in addition to the liver and contribute to inflammatory responses.

Together, these data reveal that upon LPS treatment, ER-dependent NLRP3 inflammasome and hepatocyte death pathways were induced in the livers of *ob/ob* mice, whereas TUDCA blocked both pathways. We next decided to administer the TUDCA treatment with LPS for the duration of the 6-h treatment. Importantly, we observed that a unique dose of TUDCA was still capable of protecting the liver against LPS-induced steatohepatitis foci formation and necrosis (Supplementary Figure S2A), liver injury (Supplementary Figure S2B) and apoptosis (Supplementary Figure S2C) independently of the grade of steatosis (Supplementary Figure S2A). TUDCA 6-h co-treatment decreased the activation of inflammatory caspases in the liver at the protein and mRNA levels (Supplementary Figure S3A, top and bottom, respectively). The increase in circulating levels of IL-1*β*, TNF*α* and IFN*γ* in response to LPS was also limited by TUDCA (Supplementary Figure S3B). Thus, the hepatoprotective and anti-inflammatory properties of TUDCA against LPS are independent of its ability to improve steatosis.

### TUDCA reduces LPS-induced hepatic IRE1*α* and PERK activation

**Figure 3:**
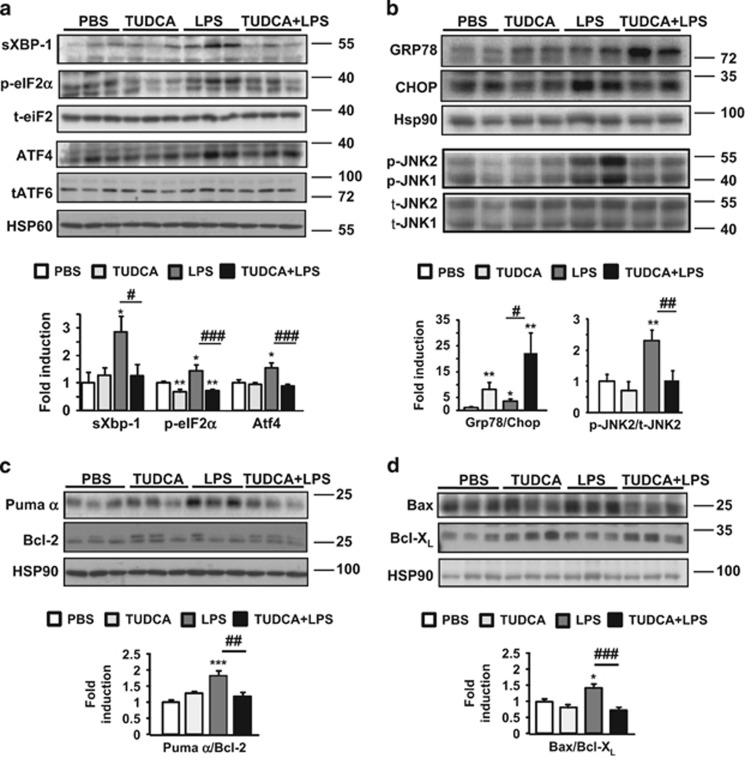
TUDCA inhibited the LPS-induced IRE1*α* and PERK hepatic activities by favoring anti-apoptotic signaling pathways in *ob/ob* mice. The expression of ER stress (**a** and **b**) and apoptotic proteins, presented as Bcl-2 family pro-apoptotic/anti-apoptotic ratios (**c** and **d**), was compared by immunoblotting and quantified (*n*=6–9). Data are expressed as mean±standard error of the mean. Statistical significance from controls is denoted by *^,#^*P*≤0.05, **^,##^*P*≤0.01, ***^,###^*P*≤0.001

During ER stress, IRE1*α* initiates an unconventional splicing of the mRNA encoding an isoform of the XBP-1 protein (sXBP-1).^5^ PERK phosphorylates eIF2*α*, which results in the translational induction of ATF4. ATF6 is cleaved and its cytosolic domain translocates to the nucleus. We examined the hepatic status of sXBP-1, phospho-eIF2*α*, ATF4 and total ATF6 in *ob/ob* mice. The levels of the sXBP-1 protein (Figure 3a) and target gene *DnaJ9* (Supplementary Figure S4a) increased significantly in LPS-treated mice, whereas they were barely detected in [TUDCA+LPS]-treated mice. In addition, the hepatic levels of phosphorylated eIF2*α* and ATF4 protein expression were slightly enhanced with LPS, while TUDCA pretreatment protected from LPS-induced eIF2*α* activation (Figure 3a). Interestingly, 5 days of TUDCA treatment reduced the basal state of phosphorylation of eIF2*α*. In contrast, total ATF6 expression remained unchanged irrespective of LPS stimulation (Figure 3a). The GRP78 protein was markedly increased in the livers of TUDCA-treated *ob/ob* mice, whereas the CHOP protein was barely detectable. TUDCA further increased the [GRP78/CHOP] ratio, thereby promoting potential protection against LPS stimulation (Figure 3b). Importantly, we found similar results on an mRNA level when TUDCA and LPS were administrated together for the duration of the 6- h treatment (Supplementary Figure S3C).

As reported,^3^ the basal levels of phospho-JNK were already elevated in the steatotic liver as a consequence of IRE1*α* activation compared with lean control livers (Supplementary Figures S5A–C). Upon LPS challenge, the levels of phospho-JNK rose further, whereas TUDCA treatment prevented exaggerated JNK activation (Figure 3b). CHOP mediates its pro-apoptotic effects by positively regulating pro-apoptotic Puma and Bax proteins, while negatively regulating the anti-apoptotic Bcl-2 protein.^5^ Importantly, LPS stimulation increased Puma and Bax protein expression (Figures 3c and d). TUDCA blocked this upregulation and slightly increased the levels of Bcl-X_L_ and Bcl-2 proteins. Thus, TUDCA treatment inhibited the increase in Bax and Puma protein levels, thereby promoting a protective Bcl-X_L_- and Bcl-2-dependent mechanism against LPS-induced liver injury.

These data revealed that in livers from obese mice, administration of LPS results in the activation of IRE1*α* and PERK, as well as CHOP overexpression. This, in turn, activates the NLRP3 inflammasome, initiating hepatocyte apoptosis and, more specifically, pyroptosis. In contrast, the LPS challenge is blocked by the ER stress inhibitor TUDCA. In light of the data, we addressed whether feeding mice with a methionine- and choline-deficient (MCD) diet, a nutritional model of steatohepatitis, would induce a similar phenotype. As expected, the MCD-fed mice developed a typical feature of NASH: increased liver injury (Supplementary Figure S6A). All these parameters correlated with an increased hepatic priming of the NLRP3 inflammasome and ER stress markers, specifically CHOP, in the MCD-fed mice (Supplementary Figures S6B and C).

### Tunicamycin treatment leads to hepatic apoptosis, exacerbated NLRP3 inflammasome activation and overwhelmed IRE1*α* and PERK activities

**Figure 4:**
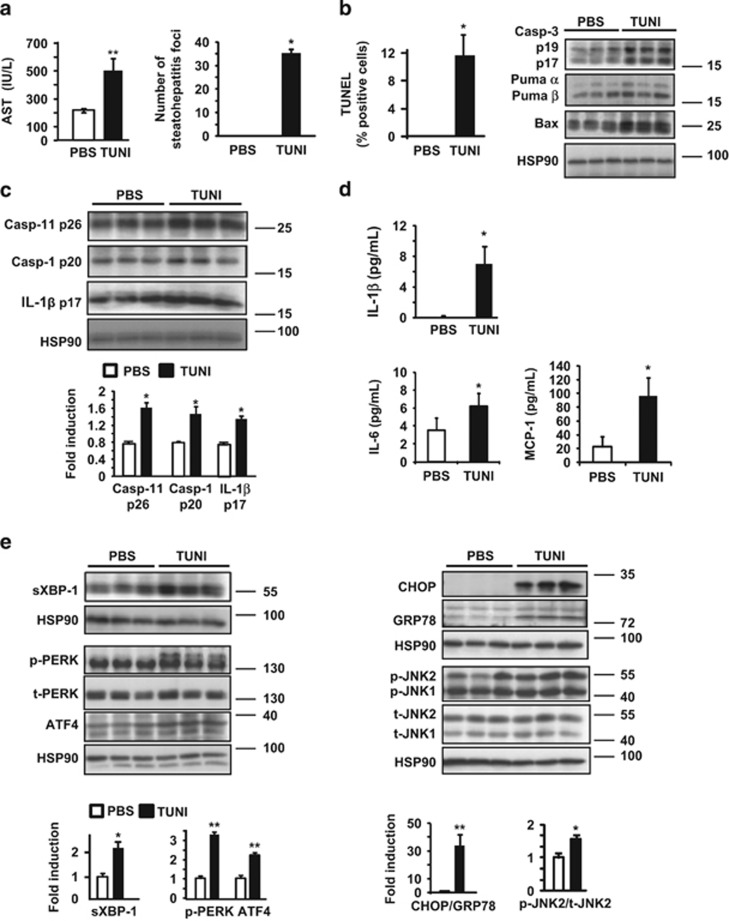
Challenge with tunicamycin in *ob/ob* mice increased apoptosis and activation of the NLRP3 inflammasome, and was associated with hepatic IRE1*α* and PERK activities. Mice were injected with TUNI (2 *μ*g/g) 6 h before killing. (**a**) Serum AST (IU/l) levels were measured and the number of steatohepatitis foci was determined (*n*=5). (**b**) The number of apoptotic hepatocytes was monitored by TUNEL staining. Immunoblotting of active caspase-3, Puma and Bax was performed from whole-liver lysates. (**c**) Immunoblot analysis of the protein levels of active caspase-11, caspase-1 and IL-1*β* are shown (*n*=5). (**d**) Plasma levels of the cytokines IL-1*β*, IL-6 and MCP-1 are represented (*n*=5). (**e**) The expression of ER stress proteins was compared in TUNI- and PBS-injected mice (*n*=5). Data are expressed as mean±standard error of the mean. Statistical significance from controls is denoted by *P≤0.05, **P≤0.01

We questioned whether ER stress activation with tunicamycin (TUNI), a specific ER stress inducer, would lead to increased liver injury and NLRP3 inflammasome activation in the livers of obese mice. As shown in Figure 4a, the serum levels of AST were significantly increased in TUNI-injected mice compared with control animals, indicative of additional hepatocyte death, and we observed a strong increase in the number of steatohepatitis foci. Furthermore, these mice present a marked increase in TUNEL-positive hepatocytes, activated hepatic caspase-3, Puma *α* and active Bax in response to TUNI (Figure 4b). These results clearly indicate that ER stress by TUNI led to liver injury associated with hepatocyte apoptosis in *ob/ob* mice.

Regarding inflammasome activation, we found that TUNI exacerbated hepatic caspase-11, caspase-1 and production of IL-1*β* compared with control mice (Figure 4c). Importantly, in agreement with increased caspase-11 and -1 activation, the serum levels of systemic mature IL-1*β* increased after TUNI injection (Figure 4d). TUNI treatment also increased the circulating levels of the proinflammatory cytokines IL-6 and MCP-1 (Figure 4d). The mRNA level of *Nlrp3* was also specifically increased with TUNI (Supplementary Figure S4B). Hence, the induction of ER stress by TUNI administration not only triggered apoptosis, but also led to an increase in hepatocyte pyroptosis in *ob/ob* mice.

We thus analyzed the activity of UPR effectors in response to TUNI. The level of sXBP-1, a target of IRE1*α*, was significantly increased after TUNI treatment (Figure 4e). Consequently, the mRNA levels of *sXBP-1* and the target gene *DnaJ9* (Supplementary Figure S4B) were increased in response to TUNI. Simultaneously, levels of hepatic phospho-PERK increased in TUNI-challenged mice compared with control mice. Accordingly, we detected a marked increase in the protein (Figure 4e) and mRNA levels (Supplementary Figure S4B) of ATF4. Finally, a strong upregulation in CHOP protein expression, a downstream target of sXBP-1 and ATF4,^5^ was detected after TUNI challenge, alongside an increase in phospho-JNK expression (Figure 4e). Accordingly, TUNI increased the [CHOP/GRP78] ratio at the protein (Figure 4e) and the mRNA levels (Supplementary Figure S4B), thus favoring programmed hepatocyte death.

### CHOP mediates ER-stress-induced pyroptosis and apoptosis in mouse primary hepatocytes

**Figure 5:**
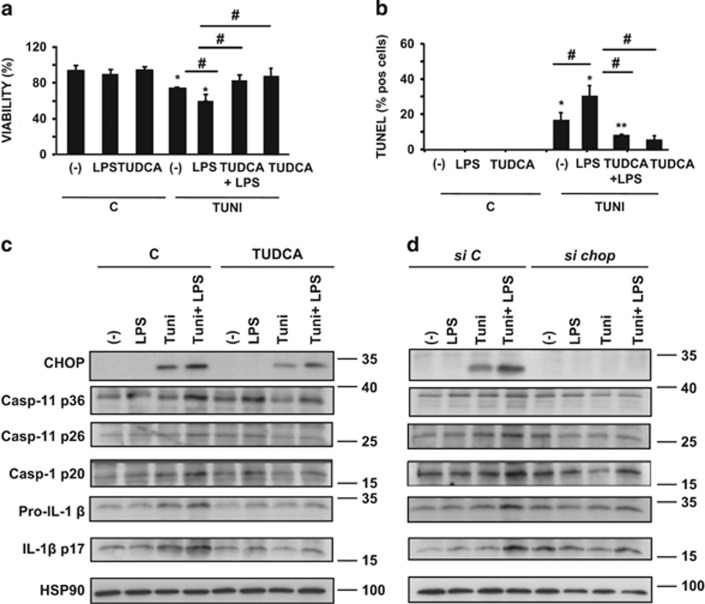
TUDCA inhibited CHOP-induced apoptosis and activation of the inflammasome in response to LPS and tunicamycin co-treatment in primary mouse hepatocytes. (**a** and **b**) Hepatocytes were pretreated for 48 h with 500 *μ*g/ml TUDCA followed by culture for 24 h in normal medium (**c**) or 100 ng/ml LPS, 1 *μ*g/ml TUNI or both (LPS+TUNI). After treatment, the percentage of viable cells quantified by MTT assay (**a**) (relative to control) and TUNEL-positive hepatocytes (**b**) was quantified (*n*=4–6). (**c** and **d**) Immunoblot analysis of CHOP, active caspase-11, caspase-1, pro-IL-1*β* and IL-1*β* protein levels assessed from hepatocytes pretreated either with TUDCA (**c**) or *Chop* siRNA (**d**) before stimulation (*n*=3). Data are expressed as mean±standard error of the mean. Statistical significance from controls is denoted by *^,#^*P*≤0.05, **P≤0.01

We evaluated cell viability, pyroptosis and apoptosis in primary mouse hepatocytes treated with LPS or TUNI, or co-treated with TUNI and LPS [TUNI+LPS], in the absence or presence of the ER stress inhibitor TUDCA. Although LPS and TUDCA alone did not alter hepatocyte viability (MTT test, Figure 5a) or positivity for TUNEL staining (Figure 5b), primary hepatocytes displayed enhanced sensitivity to co-treatment with TUNI+LPS, compared with TUNI alone, with a marked decrease in viability and a higher percentage of TUNEL-positive hepatocytes (Figures 5a and b). Furthermore, hepatocyte death induced by TUNI+LPS was partially suppressed by TUDCA. We also confirmed these results in AML12 hepatocytes (Supplementary Figure S7). We also tested whether Z-YVAD-fmk, a caspase-1 and caspase-11 inhibitor, could block the hepatocyte death induced by TUNI+LPS. The cell death caused by TUNI+LPS was indeed attenuated by Z-YVAD-fmk (Supplementary Figure S7). These results suggest that loss of viability was dependent on proinflammatory caspase-1 and caspase-11 activities. We next monitored the protein levels of proinflammatory caspases, IL-1*β* and CHOP by immunoblotting. Expression of the CHOP protein was enhanced in TUNI-treated hepatocytes, whereas it was barely detectable in controls (Figure 5c). Strikingly, CHOP protein expression was further increased in hepatocytes treated with TUNI+LPS. Importantly, this pattern of CHOP activation mirrored the increase in the active forms of caspase-11, caspase-1 and IL-1*β*. These effects were strongly inhibited by TUDCA (Figure 5c). We hypothesized that CHOP could induce the activation of inflammatory caspases, but not apoptotic caspase-3. We then performed similar experiments in primary hepatocytes by knocking-down *Chop* using siRNA. As shown in Figure 5d, the knockdown of endogenous *Chop* strongly prevented the accumulation of active caspase-11, caspase-1 and their substrate IL-1*β* in response to TUNI or TUNI+LPS, but not active caspase-3 (data not shown). This result indicated that TUNI and LPS act synergistically to induce CHOP-dependent inflammasome pyroptotic death.

### NASH patients show increased hepatic ER stress, inflammasome priming and liver injury

**Figure 6:**
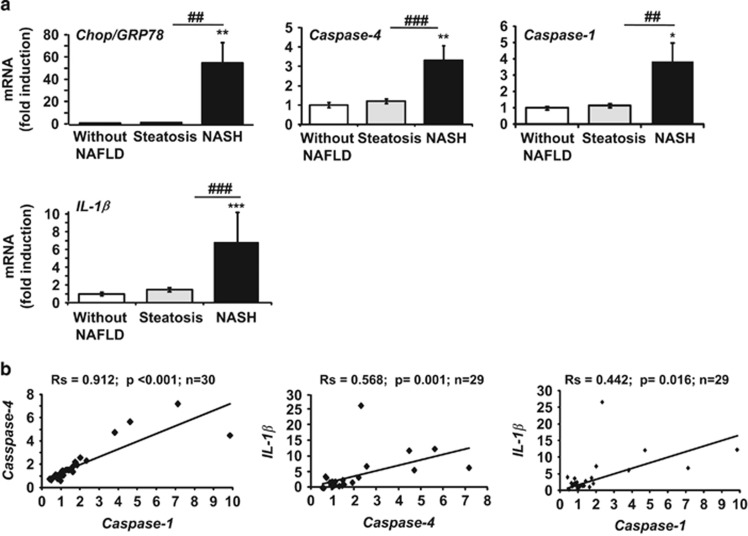
Increased expression of ER stress and inflammasome gene expression in human NASH. (**a**) The mRNA expression of *Chop*, *Grp78*, *caspase-4*, *caspase-1* and *IL-1β* (normalized to *RPLPO* mRNA) was measured in the livers of massively obese patients without NAFLD (*n*=6), with steatosis (*n*=15) or with NASH (*n*=9). Statistical significance of controls (patients without NAFLD) was determined with the Mann–Whitney test. (**b**) Correlation between NLRP3 inflammasome components at the relative mRNA expression level is shown. Correlation was evaluated using the Spearman's rank correlation test. Statistical significance from controls is denoted by *P≤0.05, **^,##^*P*≤0.01, ***^,###^*P*≤0.001

In line with our results, we hypothesized that progression from steatosis to NASH could be associated with enhanced hepatic ER stress and inflammasome activation in obese patients. This was tested by evaluating the expression of ER stress markers (*Chop* and *Grp78*) and inflammasome priming (*caspase-4*, which shares 60% homology with murine *caspase-11*), *caspase-1* and *IL-1β* in 30 obese subjects (Supplementary Table 1). The analysis of the mRNA levels showed a significant increase in both the deleterious [*Chop/Grp78*] ratio (54-fold increase), and inflammasome components (*caspase-4*, *caspase-1* and *IL-1β*) in NASH patients (*n*=9) compared with patients without NAFLD (*n*=6) and with steatosis (*n*=15, Figure 6a). These markers of ER stress and the inflammasome also correlated with the NAFLD Activity Score and liver injury, as evaluated by transaminase levels (Supplementary Table 2). In addition, we also found a positive correlation between the ER stress and level of inflammasome transcripts (Supplementary Table 2). The [*Chop/Grp78*] ratio correlated with the levels of *caspase-4* (Rs=0.414, *P*=0.029, *n*=28), *caspase-1* (Rs=0.421, *P*=0.026, *n*=28) and *IL-1β* (Rs=0.46, *P*=0.016, *n*=27) transcripts, which also correlated with each other (Figure 6b). These results indicate that ER stress and inflammasome platforms may cooperate in the progression from steatosis to NASH.

## Discussion

Steatosis is an extremely common disorder affecting nearly 30% of the US population, among which 25% develop NASH with an inherent risk for progression to cirrhosis and hepatocarcinoma. Although the function of the NLRP3 inflammasome in myeloid immune cells has been well characterized, increasing evidence shows that the NLRP3 inflammasome activation also occurs in non-myeloid cells, namely hepatocytes,^30^ in normal and pathogenic states. Nevertheless, studies have not examined whether the ER stress response stimulates hepatic NLRP3 inflammasome activation and associated cell death in NASH. Our studies suggest several potential mechanisms related to ER stress and inflammasome activation that cooperate to induce NASH development.

We first found that exaggerated ER stress obtained with LPS or TUNI in the steatotic liver leads to a transient state of NASH-like disease and the presence of hepatocyte pyroptotic cell death. Remarkably, in livers from *ob/ob* mice, challenge with LPS resulted in a degree of steatohepatitis that closely resembled human NASH, and two of the main pathological features, steatohepatitis foci and hepatocyte apoptosis, were also rapidly induced by the administration of TUNI to *ob/ob* mice. In addition, we demonstrated in these animal models that the ER stress effectors PERK and IRE1*α* converge on CHOP activation, thus increasing the activity of the NLRP3 inflammasome (caspase-11, caspase-1, IL-1*β*) and hepatic apoptosis (TUNEL positivity, caspase-3, BH3-only proteins). Second, a treatment with TUDCA dramatically reduces NLRP3 inflammasome activation and improves the NASH-pathological features in these models. Indeed, we have shown that TUDCA, administered as a protective 5-day pretreatment or as a potential 6-h treatment in *ob/ob* LPS-injected mice, exhibits anti-inflammatory and hepatoprotective properties. Third, CHOP is a critical signaling node that links ER stress-induced cell death and inflammasome activation in hepatocytes. The level of CHOP activation was robust after LPS and TUNI co-treatment, which correlated with increased cell death and activation of caspase-11 and caspase-1. Genetic silencing of *Chop* strongly reduced the activation of caspase-11, -1 and IL-1*β* production, suggesting that *Chop* modulates caspase-11 and caspase-1 activation at the transcriptional level in hepatocytes. We also observed that TUDCA protected hepatocytes from LPS- and TUNI-induced inflammasome activation and cell death, thus phenocopying the effects observed in mice with TUDCA treatment. Importantly, we reported a significant increase in gene expression of *Chop* in the livers of NASH patients, which correlated with priming of the inflammasome markers *caspase-1*, *caspase-4* and *IL-1β*. These markers significantly correlated with liver injury (transaminases) and inflammation (presence of inflammatory foci and NAS score) suggesting that the cross-talk between ER stress and inflammasome is an important mediator in the pathophysiology of NASH.

Sustained inflammasome activation can trigger apoptosis and pyroptosis, resulting in DNA damage with positivity for TUNEL staining. We found an increase in TUNEL-positive hepatocytes in both livers from LPS- and TUNI-injected mice, which correlated with increased production of active caspase-1, caspase-11, IL-1*β* and of caspase-3, PERK and IRE1*α* activities. These results suggest that LPS and TUNI induce ER-dependent pyroptosis and apoptosis in the livers of *ob/ob* mice, whereas Z-YVAD-fmk and TUDCA blocked both pathways.

Our current data demonstrate for the first time a connection between ER stress activation and the presence of pyroptotic cell death in hepatocytes with a hyperactivated NLRP3 inflammasome. Importantly, we confirmed our results in a frequently used nutritional model of steatohepatitis by feeding mice with the MCD diet.

The role of CHOP in NAFLD remains unclear, as evidence demonstrated that MCD-diet-induced steatohepatitis was reduced in *Chop* knockout mice and that inflammation was exacerbated in macrophages from *Chop*-deficient mice^13^ arguing for a cell autonomous effect of the *Chop* deficiency. Our results support a deleterious role of CHOP, driving both ER stress-induced hepatocyte death through the activation of BH3-only proteins^31^ and the NLRP3 inflammasome activation in our experimental models. Herein, we demonstrated that CHOP expression correlated with Puma and Bax induction. These results are consistent with the concept that the induction of Puma is necessary for ER stress-induced apoptosis and can be linked to direct Bax activation, initiating mitochondrial dysfunction as a downstream consequence of ER stress.^32^ These results are in accordance with the high hepatic expression levels of Puma and Bax found in patients with NASH contributing to hepatocyte lipoapoptosis.^33^ Interestingly under stress conditions, Bax and Bak can activate IRE1*α*.^34^ They could act as retro-positive controls amplifying ER stress apoptosis, inflammasome activation and downstream mitochondrial dysfunction in NASH models.

Transcriptionally, CHOP expression is regulated by ATF4, sXBP-1 and cATF6.^35^ Therefore, the increase in CHOP expression observed in the steatotic livers of mice treated with LPS and TUNI could be a reflection of both PERK and IRE1*α*/sXBP1 activation, as ATF6 remained unchanged. The increase in phospho-JNK observed in these mice could also reflect IRE1*α*/TRAF2/ASK1 activation.^5^ Interestingly, JNK is speculated to promote CHOP activity through phosphorylation,^5, 6^ thereby potentially reinforcing the PERK pathway and the IRE1*α*/sXBP-1-dependent pathway that increases CHOP production, inflammasome activation and hepatocyte death.

Such a connection between ER stress and inflammasome pathways has been recently suggested through the thioredoxin-interacting protein (TXNIP) which associates PERK and IRE1*α* with the NLRP3 inflammasome, thus activating *β*-cell death and contributing to diabetes.^36, 37^ We did not observe any variation of TXNIP protein expression in our experimental conditions suggesting that TXNIP does not seem to be a target of hepatic IRE1*α* and PERK, at least in our models (Supplementary Figure S8). Hyperactivated IRE1*α* or irremediable ER stress would also spontaneously generate ROS.^36^ We found that the level of ROS production was slightly increased after LPS and TUNI co-treatment (Supplementary Figure S8). As ROS enhance the activation of NLRP3 inflammasome, they may further amplify effects of the IRE1*α*-PERK-CHOP axes to increase pyroptosis in our experimental conditions. Future studies seeking to characterize the tight link between ER stress and NLRP3 pathways and its contribution to liver inflammation and cell death are worth considering.

As other inflammasome pathways have been described,^38^ our results support a model in which the severity of the ER stress response could activate these pathways, in hepatocytes and nonparenchymal cells, resulting in the induction of proinflammatory signaling, hepatocyte pyroptotic death and fibrosis in various liver pathologies, such as NASH, ASH and HCC. For example, AIM2 could be an attractive candidate as it senses damage-associated molecular patterns, such as cytoplasmic and mitochondrial DNA, which are increased in NASH patients.^39^ AIM2 could form an NLRP3-independent inflammasome with Pycard and caspase-1, contributing to the pathological features of these liver diseases.

**Figure 7:**
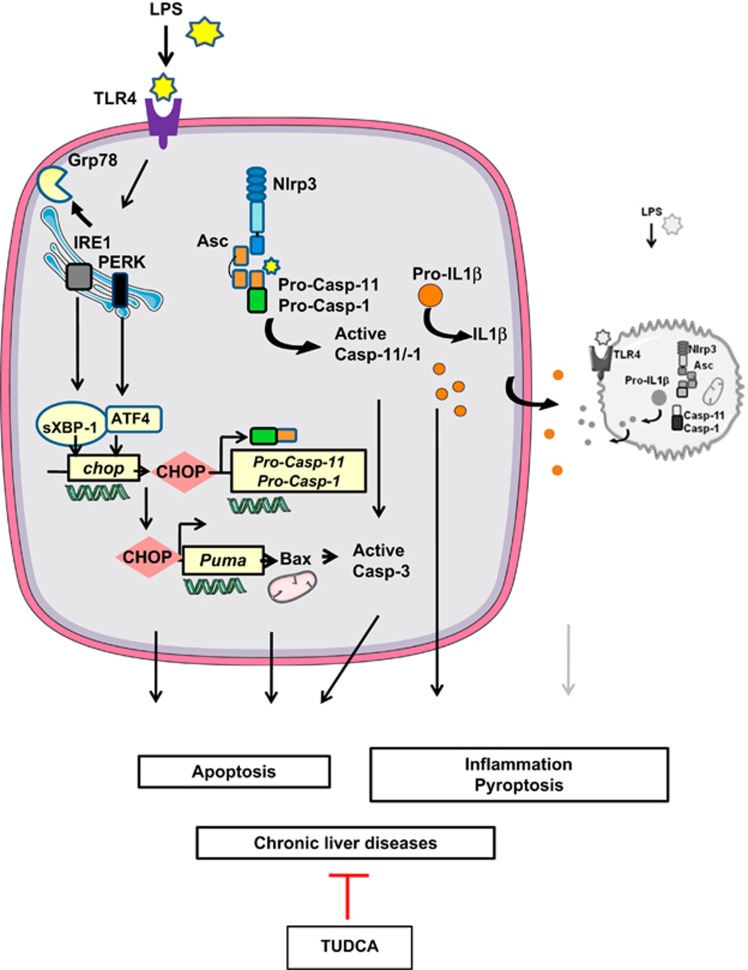
ER stress activates NLRP3 inflammasome and hepatocyte death in liver disorders. Endotoxinemia (LPS), a pathological condition found in chronic liver diseases, overwhelms hepatic IRE1*α* and PERK activities, leading to the overexpression of CHOP, which regulates the expression of *caspase-1*, *caspase-11* and Puma, and triggers hepatocyte pyroptosis and apoptosis. TUDCA increased the [GRP78/CHOP] ratio, promoting protection against ER-dependent NLRP3 inflammasome and cell death pathways. Using TUDCA, either alone or combined with hepatoprotective and anti-inflammatory interventions, could be a valid therapeutic strategy for the treatment of liver disorders

A therapeutic strategy that aims to target these common processes might be effective; TUDCA could provide such a strategy. In our animal models, it is possible that TUDCA decreases the amount of inflammatory mediators produced by activating inflammatory macrophages and the inflammatory microenvironment. Studies have reported that TUDCA decreases the amount of TNF*α* produced by inflammatory macrophages in a model of HCC.^40^ TUDCA could also decrease the expression of TLR4, the receptor of LPS. In the liver, TLR4 is expressed in both hepatocytes and immune cells such as macrophages. TLR4 specifically activates IRE1*α* to increase cytokine production (IL-6 and TNF*α*) in macrophages.^41^ Such a mechanism could also occur in hepatocytes contributing to amplify the inflammatory responses provoked by an LPS challenge (Figure 7). Further studies are needed to fully answer this question.

In summary, we have demonstrated that ER stress leads to NLRP3 inflammasome activation, thus resulting in severe liver inflammation and hepatocyte pyroptotic death, and contributing as a novel mechanism of ER-mediated liver damage (Figure 7). In this way, blocking ER-dependent NLRP3 inflammasome and cell death pathways, with TUDCA alone, or combined with other hepatoprotective and anti-inflammatory interventions, may represent a valid therapeutic strategy for the treatment of liver disorders.

## Materials and Methods

### Animal care, mouse model and treatments

All the animal procedures were conducted in compliance with protocols approved by local ethical government authorities. Male, *ob/ob* mice (C57BL/6J-ob/ob), at 6 weeks of age were purchased from Janvier Laboratories (Saint-Berthevin, France). Experiments were started 2 weeks after the arrival of the mice in our animal facility. Four different treatment protocols were administered: (1) TUDCA was injected intraperitoneally (250 *μ*g/g twice a day, total 500 *μ*g/g/day) for 5 days. Control mice received the same volume of vehicle (PBS). Mice were subjected to a single LPS injection (2 *μ*g/g) 6 h before being killed. (2) TUDCA and LPS were co-injected intraperitoneally at the above-mentioned concentrations for the duration of the 6-h treatment. Other mice were either injected with PBS, TUDCA or LPS 6 h before being killed, at the concentrations stated previously. (3) Mice were intraperitoneally injected with tunicamycin (2 *μ*g/g) or vehicle control (PBS) 6 h before being killed. All the mice were fed a normal chow diet (A04, Safe Diet, Augy, France). (4) Wild-type C57BL/6 male mice (16–18 weeks of age), from Janvier were fed a methionine- and choline-deficient diet (MCD, ref 960439) or control diet (ND) (ref 960440, MP BIO) for 2 weeks. Water was available *ad libitum*.

### Biochemical analysis and cytokine measurement

Serum levels of aspartate aminotransferase (AST) and alanine aminotransferase (ALT) were determined using a standardized UV test after activation with pyridoxal-phosphate (Roche-Hitachi analyzer, ASTPM, ALTPM, Cobas, Meylan, France). The BD Cytometric Bead Array (CBA) Mouse Inflammation Kit was used to quantitatively measure cytokines by flow cytometry as described previously.^42^

### Histological evaluation

Liver tissue specimens were fixed in 10% buffered formalin, embedded in paraffin, sectioned (5 *μ*m thick), stained with hematoxylin–eosin, and then analyzed blindly by a liver pathologist.

### TUNEL assay

Liver tissue specimens were embedded in paraffin and sectioned at 5 *μ*m for processing by the TUNEL method using a commercial kit, using DAB peroxidase substrate (Roche Molecular Biochemicals, Meylan, France) and counterstained with methyl green. Specimens were evaluated by microscopy at high power magnification ( × 100) in a blinded manner. A total of 30 random fields were counted for each TUNEL-stained tissue sample. TUNEL assays on primary hepatocytes were performed following exactly the same procedure as we previously described.^43, 44^

### *In vitro* assay for viability

Cell viability was determined by a colorimetric assay based on the ability of viable cells to reduce 3-(4,5-dimethylthiazol-2-yl)-2,5-diphenyl tetrazolium bromide (MTT) as described,^45, 46^ generating a dark blue formazan product. Dissolved MTT was added to each well of the plate and the plate was incubated at 37 °C for 1 h. The absorbance at 550 nm was measured using a microplate spectrophotometer system (ELX800, Bio-TEK Instruments, Colmar, France). Results are presented as a percentage of the control values.

### Real-time quantitative PCR analysis

Total RNA was extracted from liver tissue using an RNeasy Mini Kit (Qiagen, Courtaboeuf, France). The samples were treated with Turbo DNA-free (Applied Biosystems, Courtaboeuf, France) or RNAse-free DNAse kit (Qiagen) following the manufacturer's protocols. The quality of the isolated RNA was determined using the Agilent 2100 Bioanalyser with RNA 6000 Nano Kit (Agilent Technologies, Massy, France). Total RNA was reverse-transcribed with the High Capacity cDNA Reverse Transcription Kit (Applied Biosystems). Real-time quantitative PCR was performed using the ABI PRISM 7500/Step-One Fast Real Time PCR System following the manufacturer's protocols in C3M genomics facilities. The TaqMan gene expression assays were purchased from Applied Biosystems (Supplementary Materials and Methods). Gene expression values were normalized to the value of the housekeeping gene 36B4 (mice conditions) or RPLP0 (human conditions) and calculated on the basis of the comparative cycle threshold Ct method (22DDCt) as we described previously.^44, 46^

### Immunoblot analysis

Total liver protein was isolated from snap-frozen tissues, homogenized in detergent-containing buffer, normalized for the protein content (50 *μ*g per sample), and analyzed by SDS-PAGE (8–15% gels) immunoblotting as previously described for ER stress studies^42, 46^ and for inflammasome studies.^47^ Equal loading was assured by Ponceau S staining. Western blot analyses were performed using the primary antibodies described in Supplementary Materials and Methods. Antibody detection was accomplished using horseradish peroxidase-conjugated secondary antibodies (Supplementary Materials and Methods) and an enhanced chemiluminescence method (Amersham Biosciences, Piscataway, NJ, USA). Immunoblots were scanned, and the signals were quantified using ImageJ software.

### Cellular models and treatments

Hepatocytes from mouse liver were isolated by a two-step collagenase procedure, as we previously described.^46^ Isolated cells were resuspended in Medium I (Williams' Medium E) supplemented with 10% fetal bovine serum (PAA Laboratories, Villacoublay, France), 100 units/ml penicillin, 100 *μ*g/ml streptomycin, 2 mM l-glutamine, 0.02 UI/ml insulin (Humulin, Lilly, Fegersheim, France). Viability was evaluated by trypan blue exclusion (Sigma, St. Louis, MO, USA). Hepatocytes were incubated for 4 h at 37 °C in a humidified atmosphere with 5% CO_2_. For culture, Medium I was renewed with Medium II (a fetal bovine serum-free Medium I, supplemented instead with 0.5% bovine serum albumin). Hepatocytes were also pretreated with TUDCA (500 *μ*g/ml) for 48 h or with Z-YVAD-fmk (25 *μ*M) for 1 h in Medium II. Following these incubation times, tunicamycin (TUNI; 1 *μ*g/ml), LPS (100 ng/ml) or [TUNI+LPS] was gently added to the culture for 24 h in Medium II. AML12 hepatocytes (CRL-2254, ATCC) were cultured in medium (DMEM, 4.5 g/l glucose, 100 units/ml penicillin, 100 *μ*g/ml streptomycin and 2 mM l-glutamine) supplemented with 10% fetal bovine serum (PAA Laboratories), under 5% CO_2_ at 37 °C. The conditions for stimulation were the same.

### siRNA transfection

Primary hepatocytes were transfected with *chop (ddit3)* siRNA (MSS273951, Invitrogen, Carlsbad, CA, USA) or control siRNA (Invitrogen, Low) at 30 nM using Lipofectamine RNAiMAX (Invitrogen) according to the manufacturer's instructions. After 48 h of transfection, the cells were then treated as indicated above.

### Human studies

Morbidly obese patients (*n*=30) were recruited through the Department of Digestive Surgery and Liver Transplantation (Nice Hospital) where they underwent bariatric surgery for their morbid obesity. Bariatric surgery was indicated for these patients in accordance with the French guidelines. Exclusion criteria were: presence of a hepatitis B or hepatitis C infection, excessive alcohol consumption (>20 g/day) or another cause of chronic liver disease, as previously described.^48^ The characteristics of the study groups are given in Supplementary Table 1. Before surgery, fasting blood samples were obtained and used to measure ALT and AST aminotransaminases; glucose and insulin resistance were calculated using the homeostatic model assessment (HOMA-IR) index. Surgical liver biopsies were obtained during surgery and no ischemic preconditioning had been performed. Histopathological analysis was performed according to the scoring system of Kleiner *et al.*^49^ Four histopathological features were semi-quantitatively evaluated: grade of steatosis (0, <5% 1, 5–30% 2, >30–60% 3, >60%), lobular inflammation (0, no inflammatory foci; 1, <2 inflammatory foci per × 200 field; 2, 2–4 inflammatory foci per × 200 field; 3, >4 inflammatory foci per × 200 field), hepatocellular ballooning (0, none; 1, few ballooned cells; 2, many cells/prominent ballooning) and stage of fibrosis (from none=0, to cirrhosis=4). All the subjects gave their informed written consent to participate in this study in accordance with the French legislation regarding Ethics and Human Research (Huriet–Serusclat law). The “Comité Consultatif de Protection des Personnes dans la Recherche Biomédicale de Nice” approved the study (07/04:2003, N°03.017).

### Statistical analysis

Statistical significance of differential gene expression between the two study groups was determined using the non-parametric Mann–Whitney test with the ΔCt of each group. Correlations were analyzed using the Spearman's rank correlation test. Other data from mice and cells were statistically analyzed by Student's *t*-test or ANOVA and *post hoc* analysis for multiple group comparison. Data are expressed as mean±S.E.M. Statistical significance from controls is denoted by *P⩽0.05, **P⩽0.01, ***P⩽0.001. Following the same pattern, # denotes statistical significance between specified groups.

## Abbreviations

NAFLD: nonalcoholic fatty liver disease
NASH: nonalcoholic steatohepatitis
ER stress: endoplasmic reticulum stress
UPR: unfolded protein response
IRE1*α*: inositol-requiring enzyme 1
PERK: PKR-like ER kinase
ATF6: activating transcription factor 6
CHOP: C/EBP homologous protein
GRP78: glucose-regulated protein 78
XBP-1: X-box binding protein 1
NLRP3: NOD-like receptor family, pyrin domain containing 3
TLR-4: toll-like receptor-4
HFD: high-fat diet
MCD: methionine- and choline-deficient diet
TUDCA: tauroursodeoxycholic acid
AST and ALT: aspartate and alanine aminotransferases
ATF4: activating transcription factor 4
eIF2: eukaryotic initiation factor 2
TUNI: tunicamycin
