# Supplementary material for: ER stress induces NLRP3 inflammasome activation and hepatocyte death
Source: Cell Death Dis. 2015 Sep 10;6(9):e1879–. doi: 10.1038/cddis.2015.248 (PMC4650444; doi:10.1038/cddis.2015.248)
Supplement: Supplementary Information [file cddis2015248x1.pdf]

SUPPORTING INFORMATION

SUPPORTING MATERIALS AND METHODS

Intracellular ROS measurement

Intracellular ROS levels were detected with the fluorescent probe DCFHA-DA (Life Technologies). After 24h treatment with tunicamycin (1 or 3 µg/mL) and/or LPS (100 ng/mL) or 30 min treatment with H_2O_2 (5 mM) as a positive control, in DMEM/F12 supplemented with 100 U/mL penicillin, 100 µg/mL streptomycin, 2mM L-glutamin, 1% fetal bovine serum, cells were incubated with DCFHA-DA during 20 min following the manufacturer's instructions. Fluorescence intensity was detected with a microplate fluorometer (Fluostar OPTIMA, BMG Labtech). Data were represented as fluorescence intensity/protein concentration.

Real-time quantitative PCR analysis

The TaqMan gene expression assays were purchased from Applied: Caspase-1 (Mm00438023_m1, Hs00354836_m1); Caspase-11 (Mm00432307_m1); Caspase-12 (Mm00432324_m1); Caspase-4 (Hs01031951_m1); IL-1β (Mm00434228_m2, Hs00174097_m1); IL-18 (Mm00434225_m1);NLRP3 (Mm00840904_m1, Hs00918082_m1); TNFα (Mm00443258_m1); IFNγ (Mm01168134_m1); iNOS (Mm01309897_m1); CHOP (Mm00492097_m1; Hs01090850_m1); GRP78 (Mm00517691_m1; Hs99999174_m1); XBP1 (Mm00457359_m1); DNAJ9 (Mm01622956_m1); ATF4 (Mm00515324_m1); Mouse 36B4 (Mm99999223_gH); Human RPLP0 (Hs99999902_m1).

Immunoblot Analysis

Western blot analysis were performed using the following primary antibodies: anti-GRP78 and anti-CHOP (Santa Cruz, #13968, #7351); anti-ATF6(N) (Santa Cruz, #22799); anti-XBP-1 (Santa Cruz, #7160); anti-PERK (Santa Cruz, #13073), anti-phospho-PERK (Thr 980, Cell Signaling, #3179s), anti-phospho-eIF2a and anti-eIF2 (Cell Signaling, #9721, #9722), anti-ATF4 (Santa Cruz, #sc-200); anti-HSP90 (Cell Signaling, #4877); anti-HSP60 (Santa Cruz, sc-1722); anti-cleaved and total IL-1b

(Cell Signaling, #2021, #2022); anti-Caspase-1 (Santa Cruz, #514, #515); anti-Caspase-11 (Biolegend, 647201); anti-Caspase-12 and anti-Caspase-3 (Cell Signaling, #2202, #9662), CAD/ICAD (Santa Cruz, #sc-9066); anti-phospho- and total JNK (Cell Signaling, #9251, #9252), anti-Bax (Sigma, B8429), anti-Bcl-2 and anti-Bcl-xL (Cell Signaling, #2870, #2762), anti-Puma (Abcam, #ab54288), anti-IRE1α (Novus Biologicals, #100-2323), anti-NLRP3 (AdipoGen, #20B-0014), anti-TXNIP (MBL, #K0205-3). Antibody detection was accomplished using horseradish peroxidase-conjugated secondary antibodies: anti-mouse immunoglobulin G (Jackson, #711-035-150), anti-rabbit immunoglobulin G, (Jackson #711-035-152) or anti-Rat immunoglobulin G (Dako, #P0450).

SUPPORTING FIGURE LEGENDS

Figure S1. Five days of TUDCA treatment resolved steatosis and decreased systemic inflammatory markers in *ob/ob* mice. (A) Photomicrographs of sections of murine liver stained with H&E are represented from the 4 groups analyzed: PBS-, TUDCA-, LPS- and [TUDCA+LPS]-treated mice. The ratio of liver weight compared to full body weight was calculated (scale bar = 50 µm). Arrows identify steatohepatitis foci in each condition. The degree of steatosis (% steatosis) and necrosis (% necrosis) was blindly evaluated by the pathologist from liver sections. **(B)** Serum samples were analyzed for MCP-1 and IL-6 levels. The BD™ CBA Mouse inflammation KIT was used to measure plasma cytokine levels by flow cytometry (n = 9).

Figure S2. A unique dose of TUDCA protected against LPS-induced liver injury and apoptosis in *ob/ob* mice. TUDCA was co-administered intraperitoneally (500 µg/g) with LPS (2 µg/g) for the duration of the 6-hour treatment, prior to sacrifice. **(A)** Photomicrographs of sections of murine liver stained with H&E (scale bar = 50 µm at X200 or 25 µm at X400 magnification). The number of steatohepatitis foci (number of inflammatory foci in contact with ballooned hepatocytes) was counted and the foci representatively identified by arrows. The percentages of steatosis and necrosis were evaluated by the pathologist from liver sections (n = 5). **(B)** Serum AST and ALT transaminase levels were measured. **(C)** Apoptotic hepatocytes were visualized with the TUNEL assay (n = 5). Arrows point towards TUNEL-positive hepatocytes. Scale bar = 100 µm.

Figure S3. A unique dose of TUDCA protected against hepatic pyroptosis and ER stress seen in LPS-challenged *ob/ob* mice. (A, top) Immunoblot analysis of whole liver samples from 6-hour PBS-, TUDCA-, LPS- and [TUDCA+LPS]-treated mice was performed. Active caspase-11 and caspase-1 protein levels were evaluated in each condition (n = 3-5). **(A, bottom)** Real-time quantitative PCR analysis was performed to compare relative hepatic mRNA levels of the pyroptosis markers *caspase-1*, *caspase-11*, *IL-1β*, *IL-18* (n = 3-5). **(B)** Shown are plasma cytokine levels for IL-1β, TNFα and IFNγ (n = 3-5). **(C)** Hepatic mRNA levels of the ER stress markers *Xbp-1*, *Atf4*, *Grp78*, *Chop* are

represented (n = 3-5). Quantified mRNA levels are normalized to *36B4* mRNA and expressed as fold induction.

Figure S4. (A) TUDCA limited the overexpression of *DnaJ9* mRNA, a specific target gene of sXBP-1, in *ob/ob* mice challenged with LPS. Shown is the relative mRNA expression of hepatic *DnaJ9*, as measured by real-time quantitative PCR, after 5 days of ER stress inhibition with TUDCA. **(B) Tunicamycin enhanced hepatic gene expression of ER stress markers, such as *DnaJ9,* and *Nlrp3* in *ob/ob* mice**. Shown is the relative expression of hepatic *DnaJ9*, *sXbp-1*, *Atf4*, *Chop/Grp78* and *Nlrp3* mRNAs as measured by real-time quantitative PCR. Data were normalized relative to *36B4* mRNA and are expressed as fold induction (n = 4).

Figure S5. *Ob/ob* mice showed liver injury and inherent ER stress compared to non-obese *ob/*+ controls. (A) Photomicrographs of murine liver sections at different magnifications (X200 and X400) are represented to visualize steatosis in *ob/ob* mice compared to lean *ob/*+ controls. **(B)** In comparison to *ob/*+ mice, biological parameters highlight the increased full body weight, further reflected by the elevated liver weight/body weight ratio, in *ob/ob* mice. As shown, liver steatosis is associated with increased liver injury in *ob/ob* mice, as indicated by elevated serum AST and ALT transaminase levels (IU/L; n = 5). **(C)** Immuunoblot analysis from whole liver samples shows increased expression of ER stress markers (p-IRE1α and p-JNK) in *ob/ob* mice compared to *ob/*+ controls (n = 3-5).

Figure S6. A methionine- and choline-deficient (MCD) diet induced liver injury, hepatic inflammasome activation and increased ER stress. (A) WT mice were fed with a ND and MCD for 2 weeks prior to sacrifice. Mice serum AST (IU/L) levels were measured (n = 4-8). **(B)** Relative expression of hepatic *Caspase-1, Caspase-11, Nlrp3* and *IL-1β* mRNA were measured by real-time quantitative PCR (normalized to *36B4* mRNA). **(C)** The relative hepatic expression of ER stress markers *Chop* and *Grp78* mRNAs was compared in ND- and MCD-fed mice. Data were expressed as fold induction (n = 4-8).

Figure S7. TUDCA and Z-YVAD-fmk conferred protection from potentialization of tunicamycin-induced cell death by LPS in AML12. AML12 were pretreated for 24 hours with 500 µg/mL of TUDCA followed by culture for 24 hours in normal medium (C) or 100 ng/mL LPS, tunicamycin (TUNI) (1 µg/mL) or both (LPS+TUNI). Cells were also treated with Z-YVAD-fmk (25 µM), a caspase-1 and caspase-11 inhibitor, to evaluate LPS dependence on proinflammatory caspases. After treatment, the percentages of viable cells monitored by MTT assay **(A)** and TUNEL-positive hepatocytes (**B**) were quantified (n = 4-9).

Figure S8. (A) TXNIP showed no variation in *ob/ob* mice after inflammasome or ER stress activation. Immunoblot analyses are shown for TXNIP protein levels in whole liver samples from *ob/ob* mice treated with PBS, TUDCA, LPS and [TUDCA+LPS] (**left**) or PBS and TUNI (**right**). **(B) AML12 hepatocytes challenged with both tunicamycin and LPS showed slightly increased levels of ROS**. AML12 were treated for 24 hours with TUNI (1 or 3 µg/mL) and/or LPS (100 ng/mL). A 30 min treatment with H2O2 (5 mM) was also performed as a positive control. Intracellular ROS levels were detected with the fluorescent probe DCFHA-DA and fluorescence intensity read with a microplate fluorometer. Data were represented as fluorescence intensity/protein concentration.

SUPPORTING TABLES

	Without NAFLD	Steatosis	NASH
n	6	15	9
Age (years)	37.0 ± 6.2	34.7 ± 2.3	41.1 ± 3.6
BMI (kg/m²)	43.1 ± 0.5	44.7 ± 1.3	43.5 ± 2.3
ALT (IU/L)	14.6 ± 1.8	31.7 ± 2.0*	105.1 ± 30.6*#
AST (IU/L)	18.5 ± 1.8	22.7 ± 0.6	66.5 ± 17.3*#
Fasting insulin (mIU/L)	11.0 ± 3.3	16.6 ± 2.4	38.1 ± 7.6*#
Fasting glucose (mmol/L)	4.8 ± 0.1	5.5 ± 0.1*	9.0 ± 1.9*
HOMA-IR	2.3 ± 0.6	4.2 ± 0.7	14.4 ± 3.7*#
HbA1c (%)	5.3 ± 0.2	5.6 ± 0.1*	7.2 ± 0.7*#
**NAFLD Activity Score (n)**	**0 (6)**	**2 (6) 3 (9)**	**5 (9)**
Grade of steatosis (n)	0 (6)	2 (6) 3 (9)	3 (9)
Lobular inflammation (n)	0 (6)	0 (15)	1 (9)
Hepatocellular ballooning (n)	0 (6)	0 (15)	1 (9)

Table 1. The characteristics of obese patients. Shown are: patients with normal liver histology (Without NAFLD), patients with steatosis (Steaosis), and patients with severe steatosis and NASH (NASH). Data are compared using the non parametric Mann Whitney test: * $p < 0.05$ compared with patients Without NAFLD and # $p < 0.05$ compared with patients with Steatosis.

Fold	NAS			Inflammation			ALT			AST		
	r_s	P	N	r_s	P	N	r_s	P	N	r_s	P	N
Chop/Grp78	**0.445**	**0.016**	**29**	**0.633**	**<0.001**	**29**	**0.442**	**0.019**	**29**	**0.37**	**0.048**	**29**
Casp-1	**0.520**	**0.003**	**30**	**0.517**	**0.003**	**30**	**0.434**	**0.017**	**30**	**0.421**	**0.020**	**30**
Casp-4	**0.629**	**<0.001**	**30**	**0.609**	**<0.001**	**30**	**0.565**	**0.001**	**30**	**0.522**	**0.003**	**30**
Nlrp3	**0.449**	**0.013**	**30**	**0.416**	**0.022**	**30**	**0.290**	**0.119**	**30**	**0.352**	**0.057**	**30**
IL-1β	**0.636**	**<0.001**	**29**	**0.642**	**<0.001**	**29**	**0.474**	**0.009**	**29**	**0.499**	**0.006**	**29**

Table 2. Hepatic ER stress and inflammasome gene expression correlated positively with liver dysfunction in obese patients. Levels of liver dysfunction include steatosis, inflammation, NAFLD activity score (NAS), and plasma levels of alanine (ALT) and aspartate (AST) aminotransaminases. Correlations were analyzed using the Spearman's rank correlation test. $p < 0.05$ was considered as significant.
