# Supplementary figures and images for: ER stress induces NLRP3 inflammasome activation and hepatocyte death
Source: Cell Death Dis. 2015 Sep 10;6(9):e1879–. doi: 10.1038/cddis.2015.248 (PMC4650444; doi:10.1038/cddis.2015.248)

**A.**

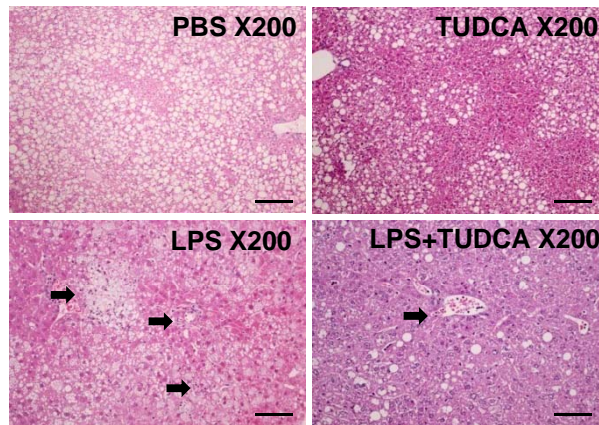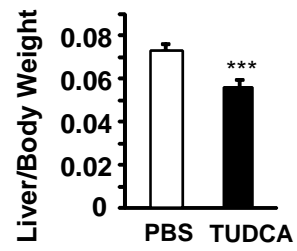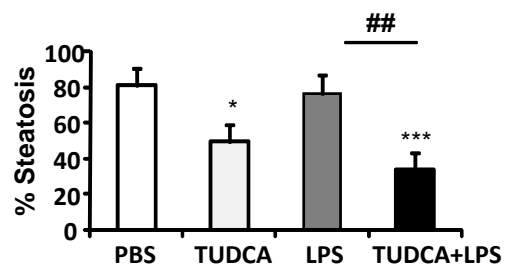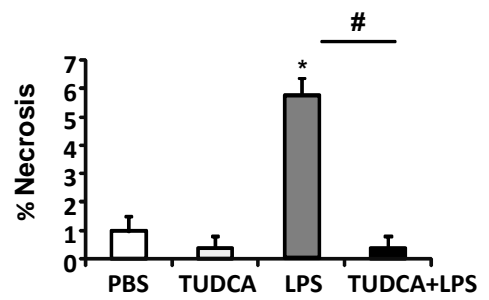

**B.**

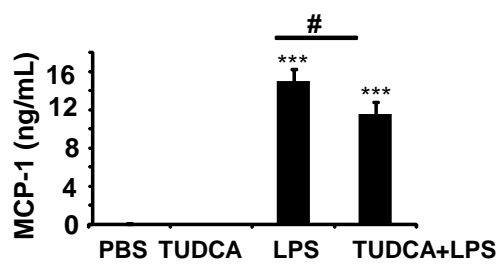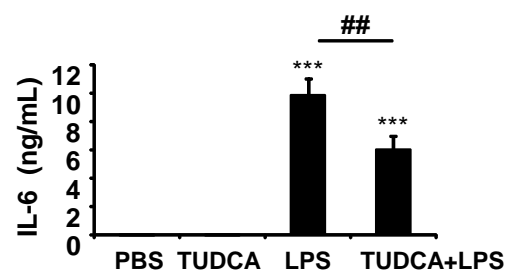

**A.**

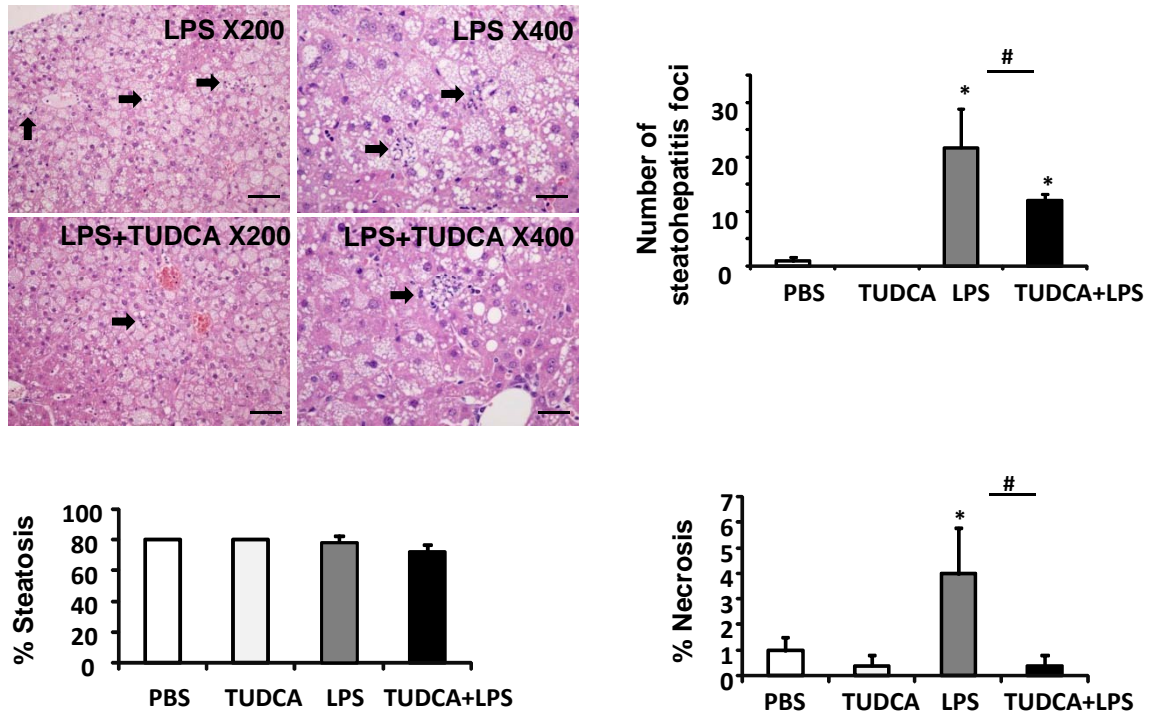

**B.**

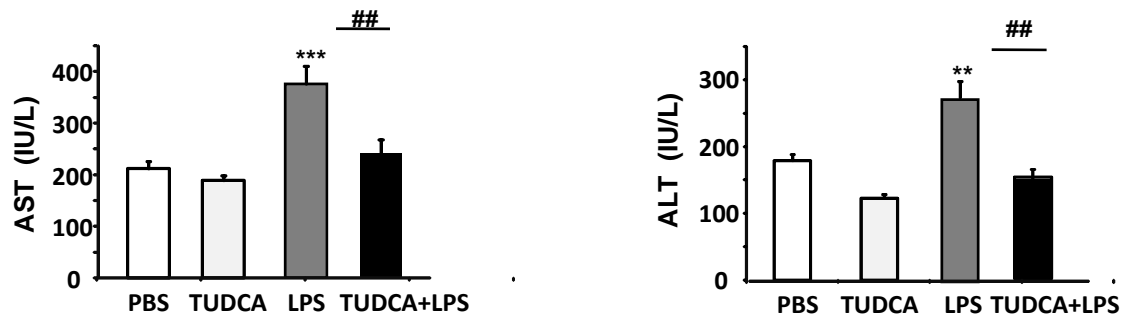

**C.**

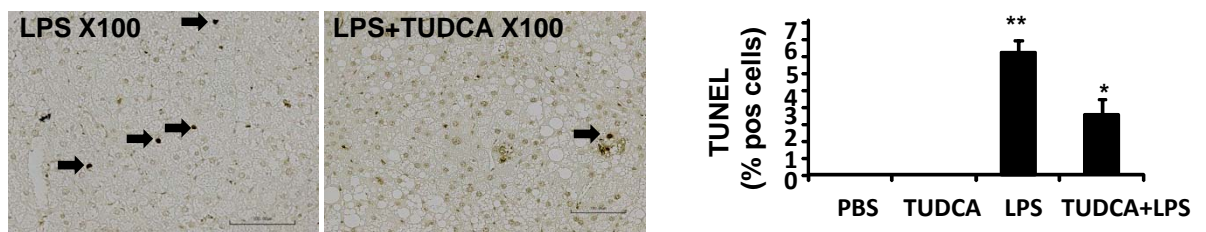

A.

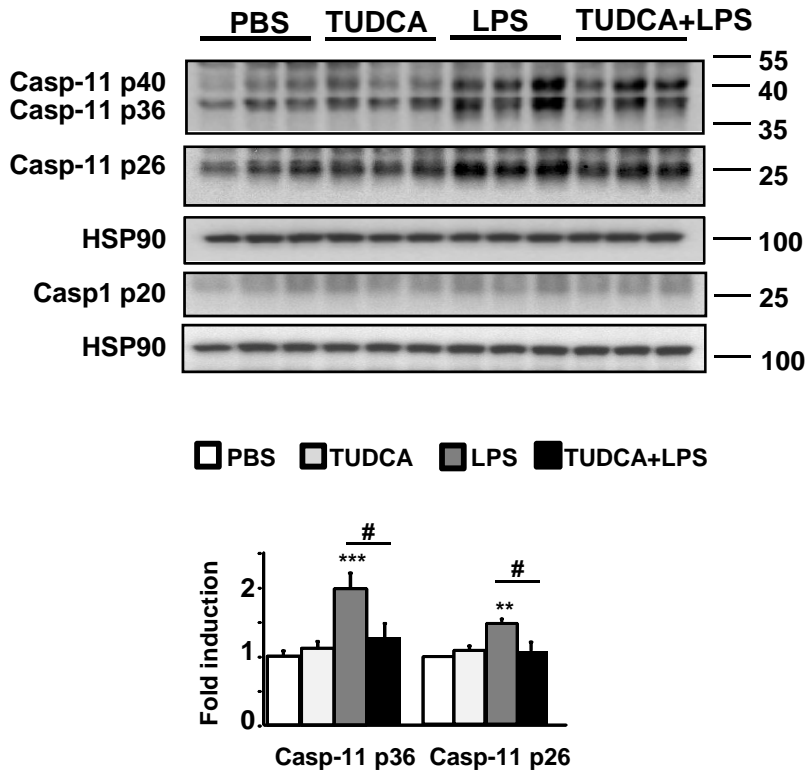

B.

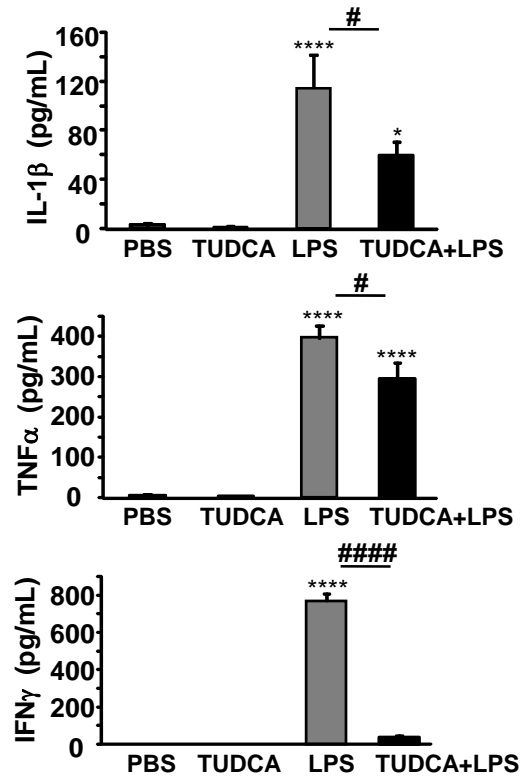

C.

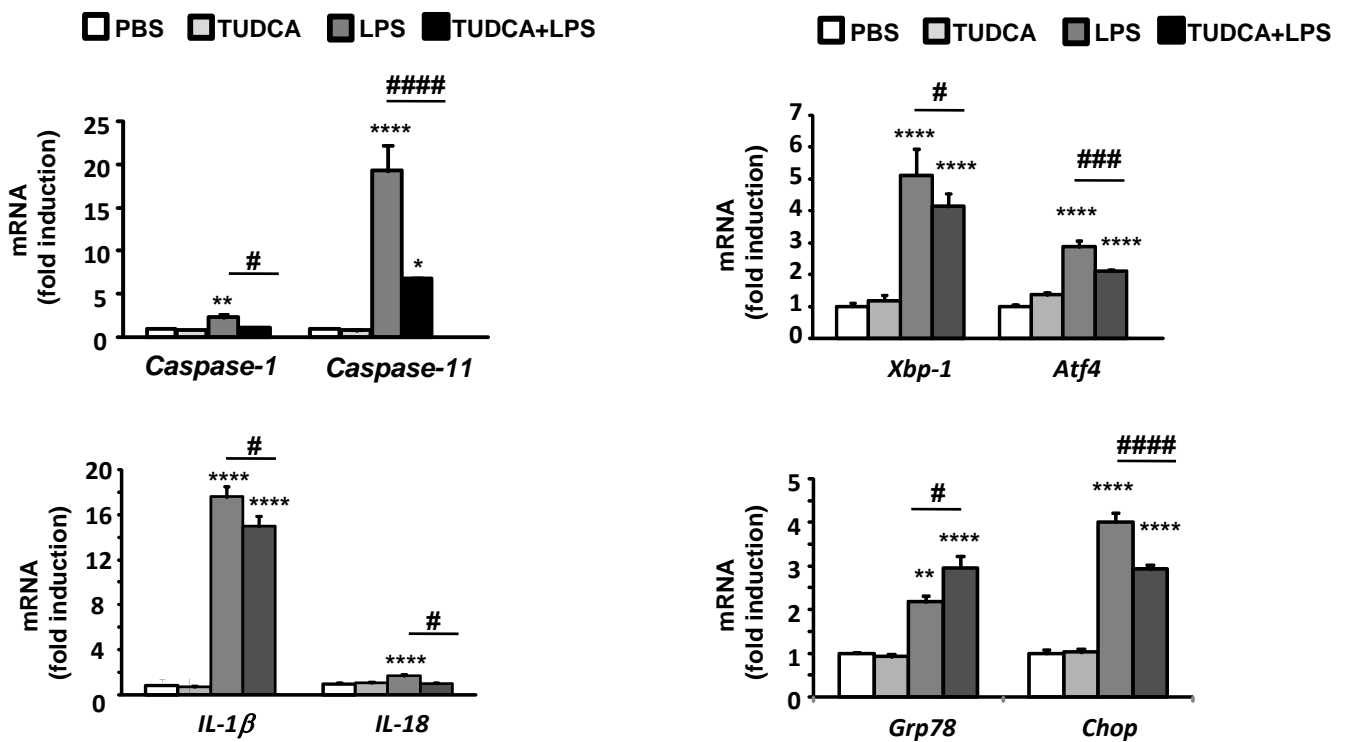

**A.**

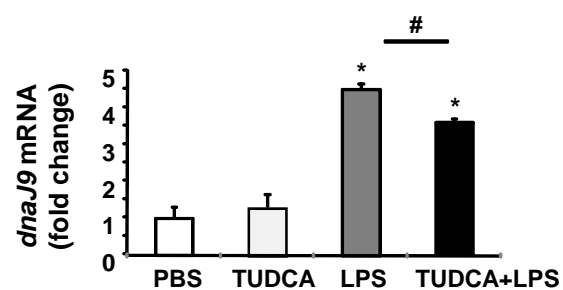

**B.**

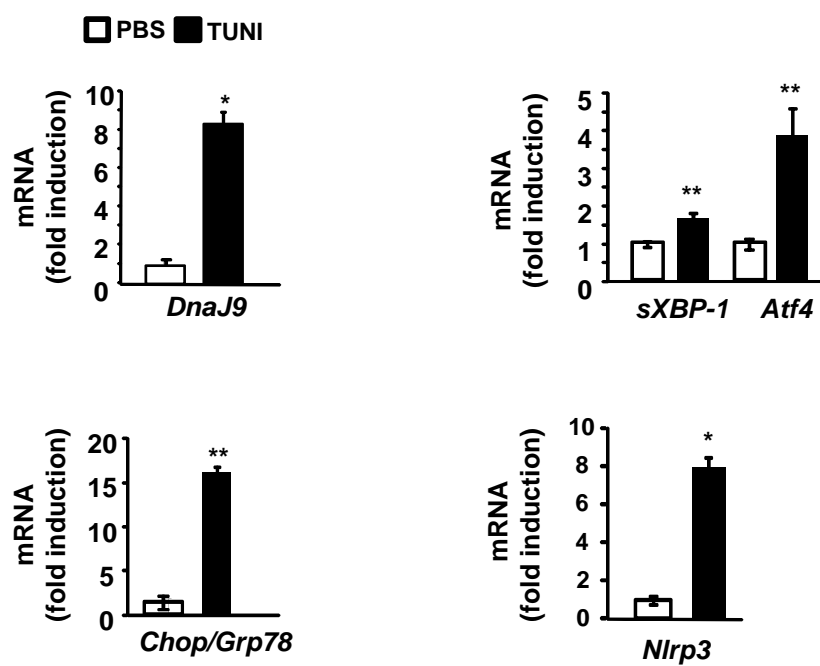

**A.**

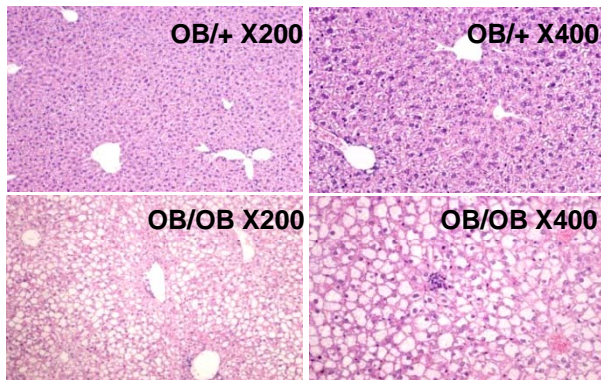

**B.**

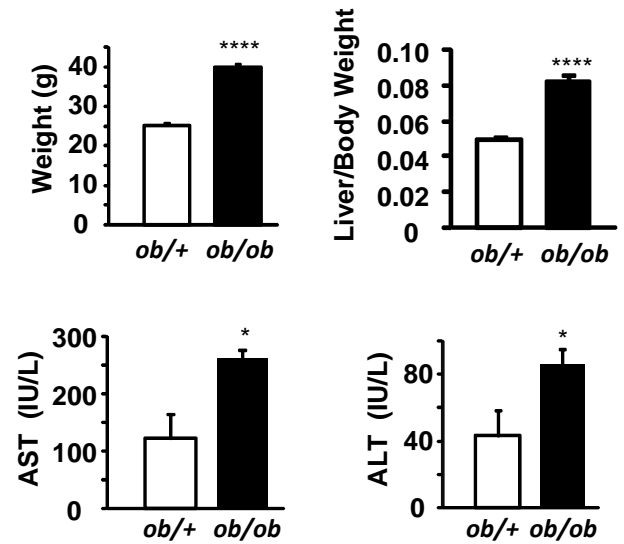

**C.**

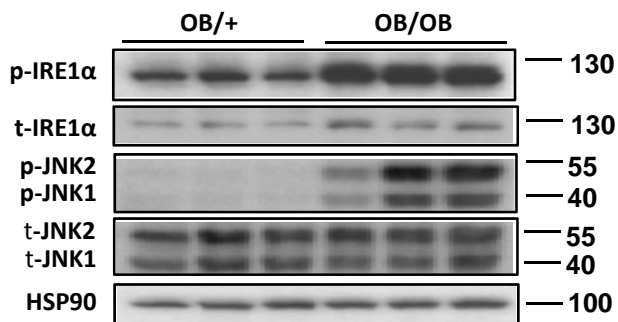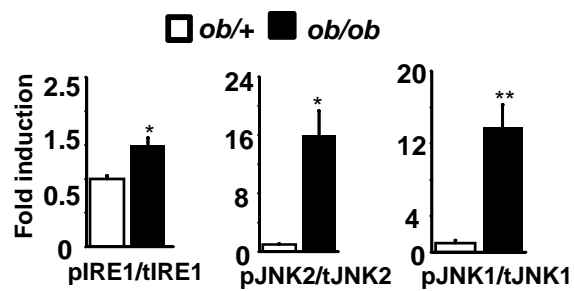

A.

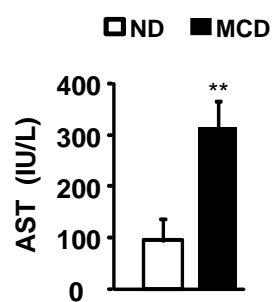

B.

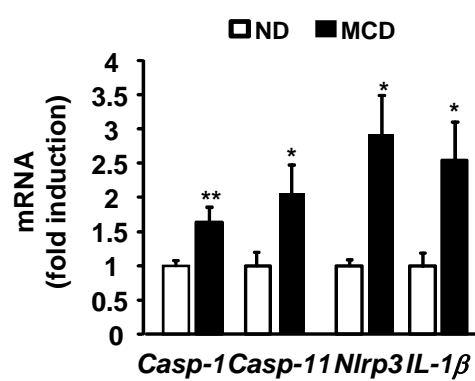

C.

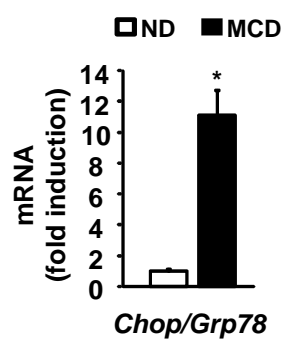

**A.**

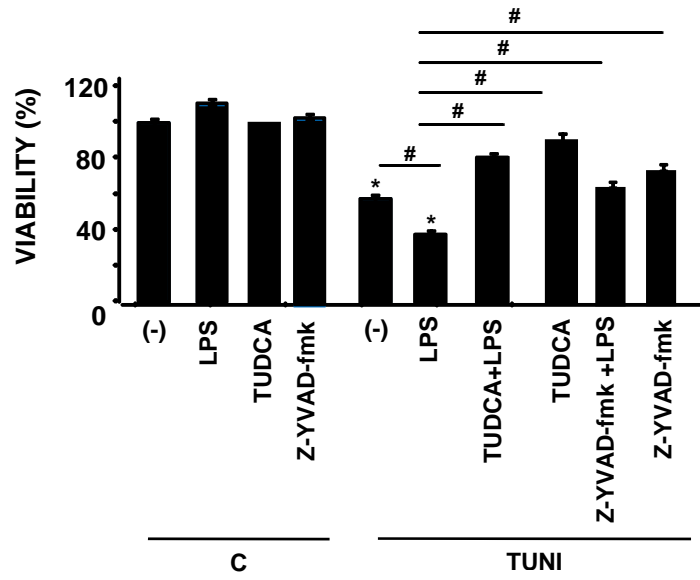

**B.**

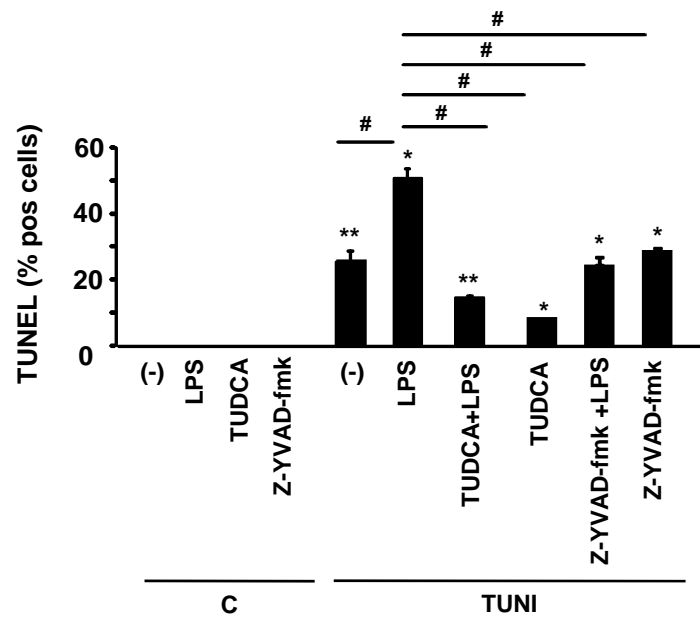

**A.**

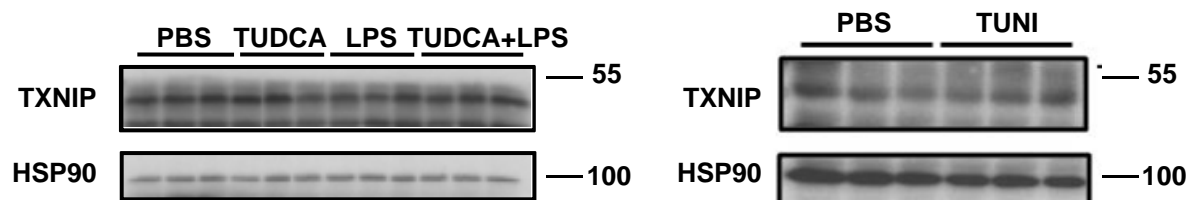

**B.**

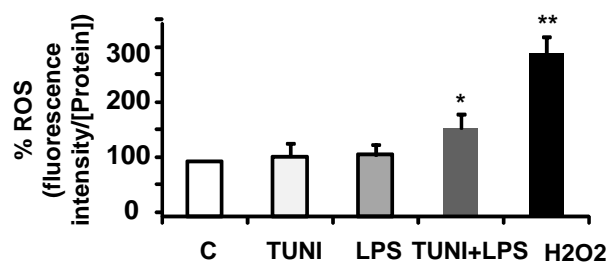

Supplement: Supplementary Figures [file cddis2015248x2.pdf]
